# Acceleration and deceleration responses to different small-sided game formats in elite youth soccer players: a repeated-measures GPS study

**DOI:** 10.3389/fphys.2026.1847447

**Published:** 2026-07-16

**Authors:** Barış Baydemir, Niyazi Eniseler, Zülbiye Kaçay, Laurentiu-Gabriel Talaghir, Gabriel Marian Manolache, Anamaria Berdila

**Affiliations:** 1 Faculty of Sports Sciences, Çanakkale Onsekiz Mart University, Çanakkale, Türkiye; 2 Faculty of Sports Sciences, Manisa Celal Bayar Universitesi, Manisa, Türkiye; 3 Faculty of Physical Education and Sport, Dunarea de Jos University of Galati, Galati, Romania

**Keywords:** acceleration, deceleration, GPS, movement demands, small-sided games, soccer

## Abstract

**Purpose:**

This study investigated the effects of different small-sided game (SSG) formats and pitch sizes on GPS-derived acceleration and deceleration event counts as indicators of external movement demands in elite youth soccer players.

**Methods:**

Twenty male elite youth soccer players (age: 19 ± 1.24 years) performed 4v1 and 6v2 SSG formats across multiple pitch dimensions (4v1: 5×5, 6×6, 7×7, and 8×8 m; 6v2: 6×6, 7×7, 8×8, 9×9, and 10×10 m). Movement data were collected from players positioned in the middle using 10 Hz GPS devices (MinimaxX v4.0, Catapult Innovations, Melbourne, Australia). Acceleration and deceleration counts were analyzed according to game format, pitch size, and bout duration (15 s and 30 s) using repeated-measures ANOVA and Holm-adjusted *post-hoc* comparisons.

**Results:**

Significant main effects of game format were observed across all acceleration and deceleration event-count variables (p < 0.001), with large effect sizes (partial η² = 0.632–0.837). In common pitch dimensions (6×6–8×8 m), the 4v1 format consistently elicited higher event counts than the 6v2 format. For example, during the 30 s condition at 6×6 m, 4v1 produced higher acceleration Zone 1 event counts than 6v2 (mean difference = 1.80; p < 0.001; Cohen’s dz = 2.24). Within the 4v1 format, the smallest pitch size (5×5 m) generated the highest acceleration and deceleration event counts, whereas in the 6v2 format the largest pitch size (10×10 m) elicited the greatest event counts.

**Conclusion:**

These findings suggest that the interaction between player configuration and spatial constraints substantially influences GPS-derived acceleration and deceleration event counts during SSGs. From a practical perspective, 4v1 SSGs performed in reduced spaces may provide an effective training stimulus for increasing acceleration and deceleration event counts. However, these outcomes should be interpreted as external movement-demand indicators and not as direct measures of quickness, agility, or perceptual-cognitive performance.

## 1 Introduction

Football is a complex, intermittent sport that requires the integration of multiple physiological, motor, technical, and tactical components to sustain performance throughout match play. Previous research has extensively examined the physical and physiological demands of football, highlighting the importance of high-intensity efforts, repeated sprint ability, and fatigue resistance during competition (Mohr et al., 2005; Fransson et al., 2017). In addition to physical capacities, successful performance in football also depends on players’ ability to self-organize, adapt to dynamic game situations, and execute tactical decisions under time pressure (Clemente et al., 2014). Therefore, performance outcomes cannot be explained solely by physical fitness, as players with similar physiological profiles may differ substantially in their tactical efficiency and decision-making ability (Delgado-Bordonau and Mendez-Villanueva, 2012).

Rapid movement actions are important components of football performance, particularly in situations requiring players to repeatedly increase and decrease running speed, adjust body position, and respond to changing task constraints. Although quickness and agility are multidimensional constructs that include perceptual, cognitive, technical, and neuromuscular components, the present study did not aim to directly measure these broader constructs (Sun et al., 2025). Instead, the study focused specifically on GPS-derived acceleration and deceleration event counts as external indicators of movement demands during small-sided games.

From a performance-monitoring perspective, acceleration and deceleration actions provide useful information about external load because they reflect rapid changes in movement velocity that may not be fully captured by distance-based measures alone. Previous studies have shown that pre-planned and reactive change-of-direction drills can improve movement speed in various sports (Bloomfield et al., 2007; Polman et al., 2004; Young et al., 2001), while small-sided game-based activities may also enhance change-of-direction performance within sport-specific contexts (Carvajal and Salazar, 2022). However, evidence remains limited regarding training approaches that integrate stimulus–response components within these contexts (Young and Rogers, 2014), and there is still a need to clarify how different possession-based SSG formats and pitch sizes influence GPS-derived acceleration and deceleration event counts in players performing a defensive role. To address this need, training methods that replicate the contextual and interactive demands of match play have gained increasing attention. Small-sided games (SSGs) are widely used in football training due to their ability to simultaneously stimulate physical, technical, and tactical adaptations (Hill-Haas et al., 2011). By manipulating task constraints such as pitch size and player numbers, coaches can modify the intensity, and movement demands of training (Brandes et al., 2012; Casamichana and Castellano, 2010). Consequently, SSGs are considered an ecologically valid training approach that aligns with the specific demands of competition while optimizing training efficiency, which is consistent with the principle of training specificity in soccer (Reilly, 2005).

Recent advances in player-tracking technologies have enabled a more detailed evaluation of external load variables during soccer training and competition. GPS-derived acceleration and deceleration metrics have emerged as important indicators of neuromuscular demands because they capture rapid changes in movement velocity that are not fully reflected by distance-based measures alone (Akenhead et al., 2013; Dalen et al., 2016). Nevertheless, evidence regarding how different SSG formats influence these variables remains limited, especially when considering specific playing roles and spatial constraints.

Despite the growing body of research on SSGs, most previous studies have primarily focused on general physical and physiological variables such as total distance covered, heart rate responses, and sprint performance (Hill-Haas et al., 2011; Castellano et al., 2013). Consequently, there is still limited understanding of how specific SSG formats influence acceleration and deceleration responses, which represent key external load indicators associated with rapid movement actions in football. Previous studies have primarily focused on general physical or physiological outputs, whereas fewer investigations have specifically examined acceleration–deceleration demands under different SSG configurations and spatial constraints. In particular, the movement demands imposed on players occupying the defensive role in possession-based SSGs remain insufficiently explored. This gap is especially relevant for designing training interventions aimed at improving rapid movement performance in football.

Therefore, the aim of the present study was to determine the most effective small-sided game format for influencing acceleration and deceleration demands associated with rapid movement actions by analyzing the acceleration and deceleration responses of players acting as defenders in 4v1 and 6v2 SSGs performed across different pitch sizes. The present study extends previous SSG research by specifically examining how the interaction between player configuration, pitch size, and defensive playing role influences acceleration and deceleration demands in elite youth soccer players.

## 2 Methods

### 2.1 Research design

This study employed a repeated-measures observational design to compare acceleration and deceleration responses of players performing defensive roles during small-sided games (SSGs) conducted in different formats and pitch sizes. All participants completed all SSG conditions under standardized training settings, allowing within-subject comparisons of movement demands across formats and spatial constraints. No experimental intervention or randomization or counterbalancing procedures were applied, as the study was conducted under regular training conditions. Data were collected under regular training conditions using global positioning system (GPS) technology to quantify movement characteristics.

### 2.2 Participants

A total of 20 male youth football players (age: 19 ± 1.24 years; height: 179.25 ± 4.63 cm; body mass: 70.85 ± 4.23 kg; BMI: 22.04 ± 0.82 kg·m⁻²) participated in the study.

Inclusion criteria were: (i) being a licensed football player, (ii) having at least five years of training experience, and (iii) being free from injury at the time of data collection. Exclusion criteria included: (i) current or recent injury and (ii) inability to complete the training protocol.

The sample size used in the present study was comparable to previous investigations examining physical and movement responses during small-sided games in soccer players (Hill-Haas et al., 2009a; Rebelo et al., 2016). An *a priori* sample size calculation was not performed. The sample size was determined based on the availability of eligible elite youth players and was consistent with sample sizes reported in previous small-sided game studies.

### 2.3 Small-sided game design

All procedures were conducted on a natural grass training pitch. Players performed SSGs in two formats:

4v1 format: 5×5 m, 6×6 m, 7×7 m, 8×8 m.

6v2 format: 6×6 m, 7×7 m, 8×8 m, 9×9 m, 10×10 m.

All participants completed all SSG conditions included in the study protocol.

In each format, the analysis focused on the players positioned in the middle (defensive role), who were required to continuously react to ball movement and attempt to regain possession.

This positional role was specifically selected because players positioned in the middle are continuously exposed to reactive movement demands and frequent acceleration–deceleration actions during possession-based SSG tasks (Hill-Haas et al., 2011; Hodgson et al., 2014). To maintain game continuity and passing quality, additional balls were placed around the pitch, allowing immediate reintroduction of play when the ball went out of bounds. Each bout lasted 15–30 seconds, and a work-to-rest ratio of 1:5 was implemented to ensure adequate recovery between repetitions (Hill-Haas et al., 2009a; Rebelo et al., 2016).

All SSG conditions were performed under similar environmental conditions and at the same time of day to minimize potential circadian and environmental influences on performance. Although detailed environmental parameters (e.g., temperature, humidity, and wind speed) were not recorded, all sessions were conducted under consistent field conditions.

Before data collection, players completed a familiarization session involving the SSG formats and GPS procedures. All trials were included in the analysis, and the mean values obtained across repetitions were used for statistical analysis.

### 2.4 Data collection

Player movements were recorded using 10 Hz GPS devices (MinimaxX v4.0, Catapult Innovations, Melbourne, Australia). Previous studies have demonstrated acceptable validity and reliability of 10 Hz GPS devices for measuring movement demands, accelerations, and decelerations in team-sport athletes (Johnston et al., 2014; Varley et al., 2012). The primary variables analyzed were the number of accelerations and decelerations performed by the players in the middle position during each SSG format. Acceleration and deceleration variables were exported from the GPS software as event counts for each SSG bout. These variables therefore represent the number of acceleration and deceleration events detected by the software during the relevant time window, rather than acceleration magnitude values expressed in m·s⁻². The software-specific algorithm and exact event-detection thresholds used to classify acceleration and deceleration events were not independently accessible to the researchers. Therefore, the present study reports the GPS-derived event-count outputs generated by the software and interprets them as external movement-demand indicators. These variables were not interpreted as direct measures of quickness, agility, reactive agility, decision-making speed, or perceptual-cognitive performance.

#### 2.4.1 Data collection tools

##### 2.4.1.1 Anthropometric measurements

Height and body mass were measured using a calibrated digital scale and stadiometer (Seca 714, Hamburg, Germany). Body mass index (BMI) was calculated as body mass (kg) divided by height squared (m²).

##### 2.4.1.2 Recording of acceleration and deceleration

Acceleration and deceleration data were recorded using the GPS system and processed with Logan Plus software (version 4.5.1, Catapult Innovations, 2010) to generate the data matrix. The exported variables consisted of acceleration and deceleration event counts recorded during each 15 s and 30 s SSG bout. These event counts were used as the primary external-load outcomes in the present study. Consistent with previous studies, speed zones were categorized into five ranges: Zone 1: 0–6.9 km·h⁻¹.

Zone 2: 7.0–12.9 km·h⁻¹.

Zone 3: 13.0–17.9 km·h⁻¹.

Zone 4: 18.0–20.9 km·h⁻¹.

Zone 5: >21 km·h⁻¹.

(Dellal et al., 2011; Hill-Haas et al., 2009a).

It is important to clarify that the zones expressed in km·h⁻¹ are speed-intensity zones and do not represent acceleration or deceleration thresholds. They were used by the GPS software to classify the speed context in which movement events occurred. Accordingly, the outcomes reported in the present study should be interpreted as acceleration and deceleration event counts classified within predefined speed-intensity zones, not as acceleration or deceleration magnitudes. Because the proprietary event-detection algorithm embedded in the software was not directly accessible, the exact acceleration/deceleration threshold values and minimum-duration criteria used by the software could not be independently verified or modified by the researchers.

### 2.5 Statistical analysis

Statistical analyses were performed using IBM SPSS Statistics (version 24). Because the same participants completed all experimental conditions, repeated-measures statistical procedures were used. Prior to the main analyses, the assumptions required for repeated-measures ANOVA were evaluated. Data normality was assessed using the Shapiro–Wilk test, and sphericity was evaluated using Mauchly’s test. When necessary, Greenhouse–Geisser corrections were applied to adjust the degrees of freedom. Potential outliers were screened prior to analysis. Two-way repeated-measures ANOVA was performed to examine the effects of game format and pitch size across common pitch dimensions (6×6, 7×7, and 8×8 m). In addition, one-way repeated-measures ANOVA was conducted to evaluate pitch-size effects within each game format separately. Holm-adjusted paired *post-hoc* comparisons were applied when significant main effects or interaction effects were identified. Effect sizes were calculated using Cohen’s *dz* for paired comparisons and partial eta squared (η²p) for repeated-measures ANOVA. Statistical significance was set at *p* < 0.05.

## 3 Results

All acceleration and deceleration outcomes are reported as GPS-derived event counts per bout. Therefore, the numerical values in the tables represent the number of detected events and do not have physical units such as m·s⁻².

Repeated-measures analyses revealed significant main effects of game format across all acceleration and deceleration variables, with large effect sizes observed for most comparisons (partial η² = 0.632–0.837). Significant interaction effects between game format and pitch size were also identified, indicating that movement demands were influenced by the combined effects of spatial constraints and player configuration.

The anthropometric characteristics of the participants are presented in **Table 1**.

**Table 1:** Anthropometric characteristics of the participants.

Variables	N	Mean ± SD	Min	Max
Age (years)	20	19 ± 1.24	17	20
Training age (years)		5 ± 0.89	5	6
Height (cm)		179.25 ± 4.63	174	185
Weight (kg)		70.85 ± 4.23	63	80
BMI (kg·m⁻²)		22.04 ± 0.82	20.05	23.09

To provide an overview of the movement demands observed across all experimental conditions, descriptive statistics for acceleration and deceleration variables according to SSG format and pitch size are presented in **Table 2**.

**Table 2:** Descriptive statistics for GPS-derived acceleration and deceleration event counts according to SSG format and pitch size.

Format	Pitch	N	15 s Acc Zone 1	15 s Acc Zone 2	15 s Dec Zone 1	15 s Dec Zone 2	30 s Acc Zone 1	30 s Acc Zone 2	30 s Dec Zone 1	30 s Dec Zone 2
4v1	5×5	20	3.05 ± 0.51	2.45 ± 0.51	2.80 ± 0.77	2.90 ± 0.72	4.25 ± 0.79	3.15 ± 0.93	4.10 ± 0.31	3.65 ± 0.59
4v1	6×6	20	2.75 ± 0.55	2.25 ± 0.55	2.65 ± 0.75	2.25 ± 0.55	3.70 ± 0.73	2.65 ± 0.49	3.65 ± 0.59	2.90 ± 0.31
4v1	7×7	20	2.40 ± 0.50	2.15 ± 0.37	2.35 ± 0.49	2.05 ± 0.22	3.35 ± 0.75	2.40 ± 0.50	3.10 ± 0.79	2.50 ± 0.51
4v1	8×8	20	2.10 ± 0.31	2.00 ± 0.32	2.05 ± 0.51	1.80 ± 0.41	2.40 ± 0.60	2.15 ± 0.59	2.40 ± 0.60	2.15 ± 0.37
6v2	6×6	20	1.55 ± 0.51	1.25 ± 0.55	1.60 ± 0.50	0.95 ± 0.39	1.90 ± 0.79	1.70 ± 0.57	1.85 ± 0.37	1.40 ± 0.82
6v2	7×7	20	1.65 ± 0.49	1.65 ± 0.49	1.90 ± 0.31	1.05 ± 0.22	2.05 ± 0.69	1.85 ± 0.37	1.95 ± 0.39	1.60 ± 0.60
6v2	8×8	20	1.85 ± 0.49	1.75 ± 0.44	1.80 ± 0.41	1.55 ± 0.51	2.10 ± 0.64	1.90 ± 0.31	2.10 ± 0.64	2.00 ± 0.32
6v2	9×9	20	2.20 ± 0.41	2.05 ± 0.22	2.20 ± 0.41	1.95 ± 0.51	2.70 ± 0.66	2.00 ± 0.46	2.30 ± 0.47	2.45 ± 0.61
6v2	10×10	20	2.55 ± 0.51	2.25 ± 0.44	2.45 ± 0.51	2.30 ± 0.47	3.40 ± 0.60	2.75 ± 0.79	3.00 ± 0.46	2.70 ± 0.57

Values are presented as mean ± SD and indicate the number of software-detected events per bout. N = 20 for each condition.

Inspection of the descriptive data revealed distinct movement-demand profiles between formats. In the 4v1 format, acceleration and deceleration values generally decreased as pitch size increased, whereas the opposite trend was observed in the 6v2 format. The highest values in the 4v1 condition were recorded on the 5×5 m pitch, while the largest values in the 6v2 condition were generally observed on the 10×10 m pitch.

The results of the two-way repeated-measures ANOVA for the common pitch dimensions (6 × 6 m, 7 × 7 m, and 8 × 8 m) are presented in **Table 3**. The two-way repeated-measures ANOVA demonstrated that game format exerted a stronger influence on movement demands than pitch size alone. Large main effects of format were observed across all variables, particularly for 15 s and 30 s deceleration Zone 2 responses (partial η² = 0.837 and 0.632, respectively). In addition, significant interaction effects indicated that the influence of pitch size differed according to SSG format.

**Table 3:** Two-way repeated-measures ANOVA for common pitch sizes (6×6, 7×7, 8×8).

Variable	Effect	*F*	df1	df2	*p*	partial η²
15 s Acc Zone 1	Format	62.986	1	19	<0.001	0.768
15 s Acc Zone 1	Pitch size	1.607	2	38	0.214	0.078
15 s Acc Zone 1	Format × Pitch size	10.116	2	38	<0.001	0.347
15 s Acc Zone 2	Format	41.988	1	19	<0.001	0.688
15 s Acc Zone 2	Pitch size	3.019	2	38	0.061	0.137
15 s Acc Zone 2	Format × Pitch size	7.733	2	38	0.001	0.289
15 s Dec Zone 1	Format	50.854	1	19	<0.001	0.728
15 s Dec Zone 1	Pitch size	1.890	2	38	0.166	0.090
15 s Dec Zone 1	Format × Pitch size	5.794	2	38	0.006	0.234
15 s Dec Zone 2	Format	97.664	1	19	<0.001	0.837
15 s Dec Zone 2	Pitch size	3.880	2	38	0.029	0.170
15 s Dec Zone 2	Format × Pitch size	15.792	2	38	<0.001	0.454
30 s Acc Zone 1	Format	67.235	1	19	<0.001	0.780
30 s Acc Zone 1	Pitch size	8.423	2	38	0.001	0.307
30 s Acc Zone 1	Format × Pitch size	15.373	2	38	<0.001	0.447
30 s Acc Zone 2	Format	37.574	1	19	<0.001	0.664
30 s Acc Zone 2	Pitch size	2.399	2	38	0.105	0.112
30 s Acc Zone 2	Format × Pitch size	6.891	2	38	0.003	0.266
30 s Dec Zone 1	Format	65.771	1	19	<0.001	0.776
30 s Dec Zone 1	Pitch size	7.238	2	38	0.002	0.276
30 s Dec Zone 1	Format × Pitch size	14.822	2	38	<0.001	0.438
30 s Dec Zone 2	Format	32.681	1	19	<0.001	0.632
30 s Dec Zone 2	Pitch size	5.715	2	38	0.007	0.231
30 s Dec Zone 2	Format × Pitch size	5.812	2	38	0.006	0.234

Within-subject factors: game format (4v1 vs 6v2) and pitch size (6×6, 7×7, 8×8). Effect size is reported as partial eta squared (η²p). To examine the independent and combined effects of game format and pitch size on acceleration and deceleration responses, a two-way repeated-measures ANOVA was conducted for the common pitch dimensions (6×6 m, 7×7 m, and 8×8 m).

*Post-hoc* comparisons showed that the 4v1 format consistently elicited greater acceleration and deceleration demands than the 6v2 format at comparable pitch sizes. The largest between-format differences were observed at the 6×6 m pitch during the 30 s condition, with very large effect sizes (Cohen’s *dz* > 2.0). Detailed Holm-adjusted pairwise comparisons between the 4v1 and 6v2 formats at the common pitch sizes are presented in **Table 4**.

**Table 4:** Paired *post-hoc* comparisons between formats at common pitch sizes.

Variable	Pitch	Mean difference	95% CI	*p*_adj	Cohen’s *dz*
15 s Acc Zone 1	6×6	1.20	0.87 to 1.53	<0.001	1.725
15 s Acc Zone 1	7×7	0.75	0.38 to 1.12	<0.001	0.954
15 s Acc Zone 2	6×6	1.00	0.63 to 1.37	<0.001	1.286
15 s Acc Zone 2	7×7	0.50	0.20 to 0.80	0.006	0.718
15 s Dec Zone 1	6×6	1.05	0.60 to 1.50	<0.001	1.132
15 s Dec Zone 1	7×7	0.45	0.12 to 0.78	0.022	0.571
15 s Dec Zone 2	6×6	1.30	0.84 to 1.76	<0.001	1.666
15 s Dec Zone 2	7×7	1.00	0.79 to 1.21	<0.001	2.236
30 s Acc Zone 1	6×6	1.80	1.28 to 2.32	<0.001	2.236
30 s Acc Zone 1	7×7	1.30	0.79 to 1.81	<0.001	1.618
30 s Acc Zone 2	6×6	0.95	0.55 to 1.35	<0.001	1.255
30 s Acc Zone 2	7×7	0.55	0.24 to 0.86	0.004	0.771
30 s Dec Zone 1	6×6	1.80	1.42 to 2.18	<0.001	2.924
30 s Dec Zone 1	7×7	1.15	0.70 to 1.60	<0.001	1.633
30 s Dec Zone 2	6×6	1.50	1.05 to 1.95	<0.001	1.666
30 s Dec Zone 2	7×7	0.90	0.50 to 1.30	<0.001	1.195

Comparison is presented as 4v1 minus 6v2. Holm-adjusted p values are reported. Following the identification of significant format effects and interactions, Holm-adjusted paired *post-hoc* comparisons were performed to determine specific differences between the 4v1 and 6v2 formats at each common pitch size (**Table 4**).

As shown in **Table 5**, separate one-way repeated-measures ANOVAs demonstrated significant pitch-size effects within both SSG formats. Within-format analyses demonstrated opposite spatial-response patterns between SSG formats. In the 4v1 format, movement demands generally increased as pitch size decreased, whereas in the 6v2 format acceleration and deceleration responses increased progressively with larger pitch dimensions. The significant pairwise comparisons identified within each SSG format across different pitch sizes are summarized in **Table 6**.

**Table 5:** One-way repeated-measures ANOVA for pitch-size effects within each format.

Format	Variable	*F*	df1	df2	*p*	partial η²
4v1	15 s Acceleration Zone 1	14.164	3	57	<0.001	0.427
4v1	15 s Acceleration Zone 2	4.457	3	57	0.007	0.190
4v1	15 s Deceleration Zone 1	5.703	3	57	0.002	0.231
4v1	15 s Deceleration Zone 2	18.728	3	57	<0.001	0.496
4v1	30 s Acceleration Zone 1	24.996	3	57	<0.001	0.568
4v1	30 s Acceleration Zone 2	8.237	3	57	<0.001	0.302
4v1	30 s Deceleration Zone 1	31.993	3	57	<0.001	0.627
4v1	30 s Deceleration Zone 2	42.311	3	57	<0.001	0.690
6v2	15 s Acceleration Zone 1	13.887	4	76	<0.001	0.422
6v2	15 s Acceleration Zone 2	14.241	4	76	<0.001	0.428
6v2	15 s Deceleration Zone 1	11.334	4	76	<0.001	0.374
6v2	15 s Deceleration Zone 2	34.762	4	76	<0.001	0.647
6v2	30 s Acceleration Zone 1	17.726	4	76	<0.001	0.483
6v2	30 s Acceleration Zone 2	11.912	4	76	<0.001	0.385
6v2	30 s Deceleration Zone 1	19.481	4	76	<0.001	0.506
6v2	30 s Deceleration Zone 2	17.288	4	76	<0.001	0.476

Within-subject factor: pitch size. Effect size is reported as partial eta squared (η²p). To further explore the influence of spatial constraints within each SSG format, separate one-way repeated-measures ANOVAs were conducted for pitch-size effects in the 4v1 and 6v2 formats (**Table 5**).

**Table 6:** Significant *post-hoc* pitch-size comparisons within each format.

Format	Variable	Comparison	Mean difference	95% CI	*p*_adj
4v1	15 s Acc Zone 1	5×5 vs 7×7	0.65	0.30 to 1.00	0.004
4v1	15 s Acc Zone 1	5×5 vs 8×8	0.95	0.63 to 1.27	<0.001
4v1	15 s Acc Zone 1	6×6 vs 8×8	0.65	0.34 to 0.96	0.002
4v1	15 s Dec Zone 2	5×5 vs 6×6	0.65	0.22 to 1.08	0.011
4v1	15 s Dec Zone 2	5×5 vs 7×7	0.85	0.52 to 1.18	<0.001
4v1	15 s Dec Zone 2	5×5 vs 8×8	1.10	0.71 to 1.49	<0.001
4v1	30 s Dec Zone 1	5×5 vs 8×8	1.70	1.21 to 2.19	<0.001
4v1	30 s Dec Zone 2	5×5 vs 8×8	1.50	1.07 to 1.93	<0.001
6v2	15 s Acc Zone 1	6×6 vs 10×10	-1.00	-1.38 to -0.62	<0.001
6v2	15 s Acc Zone 2	6×6 vs 10×10	-1.00	-1.36 to -0.64	<0.001
6v2	15 s Dec Zone 2	6×6 vs 10×10	-1.35	-1.83 to -0.87	<0.001
6v2	30 s Acc Zone 1	6×6 vs 10×10	-1.50	-2.02 to -0.98	<0.001
6v2	30 s Dec Zone 1	6×6 vs 10×10	-1.15	-1.52 to -0.78	<0.001
6v2	30 s Dec Zone 2	6×6 vs 10×10	-1.30	-1.83 to -0.77	<0.001

Significant pairwise pitch-size comparisons identified by Holm-adjusted *post-hoc* analyses are presented in **Table 6**.

## 4 Discussion

The present study examined how different small-sided game (SSG) formats and pitch sizes influence acceleration and deceleration event counts in elite youth soccer players. These variables were interpreted as indicators of external movement demands rather than direct measures of quickness, agility, or perceptual-cognitive performance. The main findings indicate that:

- the 4v1 format elicits greater acceleration and deceleration demands than the 6v2 format when played on comparable pitch sizes,
- decreasing pitch size increases movement demands in the 4v1 format, and.
- increasing pitch size leads to higher movement demands in the 6v2 format.

These findings provide further evidence that task constraints may influence neuromuscular demands in football-specific training environments. The present findings are also consistent with ecological dynamics and constraint-led approaches, which propose that movement behaviors emerge from the interaction between task, environmental, and performer constraints (Davids et al., 2013). Manipulating player numbers and pitch dimensions alters the affordances available to players, thereby influencing movement patterns and physical outputs. In the current study, the observed differences in acceleration and deceleration demands appear to reflect these adaptive responses to changing spatial and tactical constraints.

The results are consistent with previous literature demonstrating that SSG configurations significantly influence physical and neuromuscular load. For example, Rebelo et al. (2016) reported higher acceleration and deceleration outputs in smaller-sided formats compared to larger formats, suggesting that reduced player numbers increase movement intensity. Similarly, Castellano et al. (2013) showed that both pitch dimensions and player numbers are key determinants of movement profiles and intensity distribution in SSGs.

In addition, earlier studies have highlighted the role of task constraint manipulation in shaping movement behavior. Hill-Haas et al. (2009b) demonstrated that SSG formats influence sprint activity and movement characteristics, while Clemente et al. (2014) reported that physiological and performance responses vary depending on game format. Likewise, Randers et al. (2014) observed that although internal load indicators such as heart rate may remain similar across formats, external load variables—particularly acceleration-related metrics—are highly sensitive to changes in game design.

A key contribution of the present study is the identification of the interaction between player role and spatial constraints as a determinant of movement demands. In the 4v1 format, the presence of a single defender within a confined space necessitates continuous reactive adjustments, leading to frequent acceleration and deceleration actions as pitch size decreases. This finding aligns with Hodgson et al. (2014), who reported that smaller pitch dimensions increase the frequency of rapid movement changes and technical actions. Furthermore, rule-based manipulations, as demonstrated by Giménez et al. (2018), can further amplify acceleration demands, emphasizing the importance of representative task design.

In contrast, the 6v2 format presents a different movement dynamic due to the presence of two defenders. As pitch size increases, defenders are required to cover larger spatial areas and respond to broader ball circulation patterns. This may increase acceleration and deceleration demands, suggesting that spatial expansion could play an important role in shaping movement intensity within this format. These findings highlight that the interaction between spatial constraints and player configuration plays a critical role in determining external load.

Importantly, the magnitude of the observed differences, supported by large effect sizes, suggests that these variations are not only statistically significant but also practically meaningful. Previous GPS-based investigations have similarly demonstrated that acceleration and deceleration metrics are particularly sensitive indicators of external load during soccer-specific activities (Akenhead et al., 2013; Dalen et al., 2016). However, these findings should be interpreted with methodological caution because the present analysis relied on software-derived event counts rather than independently defined acceleration or deceleration thresholds. Consequently, the results describe differences in GPS/software-detected acceleration and deceleration events between SSG conditions, rather than absolute acceleration or deceleration magnitudes. Unlike total distance covered, acceleration-related variables may better capture the intermittent and explosive nature of soccer performance. Therefore, the substantial differences observed between SSG formats in the present study highlight the practical value of using acceleration and deceleration measures when monitoring training load and designing soccer-specific conditioning programs. This reinforces the notion that manipulating pitch size and player configuration can substantially alter neuromuscular demands in training.

From a practical perspective, these findings provide clear guidance for coaches and practitioners. Coaches seeking to increase acceleration and deceleration demands may preferentially use 4v1 formats performed in smaller pitch dimensions. Conversely, when the objective is to expose defenders to greater spatial coverage demands, 6v2 formats performed on larger pitches may be more appropriate. However, the present findings should not be interpreted as direct evidence of improvements in agility, reactive agility, decision-making speed, or overall quickness performance, as these variables were not directly assessed. Therefore, the practical implications of the present study are limited to the manipulation of external acceleration and deceleration demands during SSG design. These findings also support the concept of representative learning design, suggesting that training tasks that closely simulate game-specific constraints may enhance the transfer of performance adaptations to match play.

## 5 Limitations

Several limitations of this study should be acknowledged. First, the sample consisted of male elite youth players from a single team, which may limit the generalizability of the findings to other populations, including female players, younger athletes, senior players, and players competing at different performance levels. Future studies should examine whether similar acceleration and deceleration responses are observed across different age groups, sexes, and competitive standards. In addition, no *a priori* power analysis was conducted; therefore, the adequacy of the sample size should be interpreted with caution.

Second, only external load variables derived from GPS technology were analyzed. Internal load indicators, such as heart rate, blood lactate, or perceived exertion, were not included, which may limit the interpretation of physiological stress. Therefore, the present findings should be interpreted as indicators of external movement demands rather than direct measures of physiological responses.

In addition, although 10 Hz GPS technology is widely used in team sports research, potential measurement errors related to satellite signal quality and rapid movement detection should be acknowledged. Detailed GPS signal-quality indicators, such as satellite count and HDOP values, were not recorded and therefore could not be reported. Moreover, acceleration and deceleration variables were obtained as software-derived event counts. The proprietary event-detection algorithm, including the exact acceleration/deceleration thresholds and minimum-duration criteria, was not independently accessible to the researchers. This limits reproducibility and should be considered when interpreting the findings. Furthermore, technical and tactical performance variables were not analyzed, which may limit a more comprehensive interpretation of SSG demands.

Third, the analysis focused exclusively on players positioned in the middle (defensive role), and therefore the results may not be directly transferable to other playing roles within SSG contexts.

Future research should include larger and more diverse samples, integrate internal load, technical, and tactical variables, and further examine positional-role differences across different SSG formats and competitive levels.

## 6 Conclusion

In conclusion, the findings of the present study suggest that both SSG format and pitch size influence GPS-derived acceleration and deceleration event counts in elite youth soccer players. The 4v1 format performed in smaller pitch sizes produced higher acceleration and deceleration event counts, whereas the 6v2 format elicited higher event counts when performed in larger pitch sizes.

From an applied perspective, 4v1 small-sided games performed in constrained spaces may be useful when the training objective is to increase acceleration and deceleration event counts. These findings should not be interpreted as direct evidence of improved quickness, agility, or perceptual-cognitive performance. The results should also be interpreted within the methodological limits of GPS/software-derived event-count data.

## Data Availability

The original contributions presented in the study are included in the article/supplementary material. Further inquiries can be directed to the corresponding authors.
